# Differential gene expression analysis of HNSCC tumors deciphered tobacco dependent and independent molecular signatures

**DOI:** 10.18632/oncotarget.27249

**Published:** 2019-10-22

**Authors:** Inayatullah Shaikh, Afzal Ansari, Garima Ayachit, Monika Gandhi, Priyanka Sharma, Shivarudrappa Bhairappanavar, Chaitanya G. Joshi, Jayashankar Das

**Affiliations:** ^1^ Gujarat Biotechnology Research Centre (GBRC), Department of Science and Technology (DST), Government of Gujarat, Gandhinagar 382011, India

**Keywords:** tobacco, head and neck squamous cell carcinoma (HNSCC), differentially expressed genes, hub gene, miRNA

## Abstract

Head and neck cancer is the sixth most common cancer worldwide, with tobacco as the leading cause. However, it is increasing in non-tobacco users also, hence limiting our understanding of its underlying molecular mechanisms. RNA-seq analysis of cancers has proven as effective tool in understanding disease etiology. In the present study, RNA-Seq of 86 matched Tumor/Normal pairs, of tobacco smoking (TOB) and non-smokers (N-TOB) HNSCC samples analyzed, followed by validation on 375 similar datasets. Total 2194 and 2073 differentially expressed genes were identified in TOB and N-TOB tumors, respectively. GO analysis found muscle contraction as the most enriched biological process in both TOB and N-TOB tumors. Pathway analysis identified muscle contraction and salivary secretion pathways enriched in both categories, whereas calcium signaling and neuroactive ligand-receptor pathway was more enriched in TOB and N-TOB tumors respectively. Network analysis identified muscle development related genes as hub node i. e. ACTN2, MYL2 and TTN in both TOB and N-TOB tumors, whereas EGFR and MYH6, depicts specific role in TOB and N-TOB tumors. Additionally, we found enriched gene networks possibly be regulated by tumor suppressor miRNAs such as hsa-miR-29/a/b/c, hsa-miR-26b-5p etc., suggestive to be key riboswitches in regulatory cascade of HNSCC. Interestingly, three genes PKLR, CST1 and C17orf77 found to show opposite regulation in each category, hence suggested to be key genes in separating TOB from N-TOB tumors. Our investigation identified key genes involved in important pathways implicated in tobacco dependent and independent carcinogenesis hence may help in designing precise HNSCC diagnostics and therapeutics strategies.

## INTRODUCTION

Head and Neck Squamous Cell Carcinoma (HNSCC) ranks 6th, amongst the most common cancers in the world, contributing to about 5% of all malignancies globally with a death rate of nearly half of total cases [1–3]. Tobacco usage such as smoking and chewing is the most common cause of head & neck and other cancers [4, 5]. Nonetheless, there are occurrences where individuals who never used tobacco or take liquor developed malignancy [6–9].

In the past decade DNA sequencing technologies such as Next generation sequencing technology (NGS) has identified key genomic signatures involved in cancer development and progression [10–14]. In recent years, large consortium based studies such as The Cancer Genome Atlas (TCGA) has provided detailed molecular map of HNSCC, and improved improved understanding of the role of genes in the pathogenesis of HNSCC [15–17]. Similarly, several studies has demonstrated the role of RNA-sequencing technique in cancers including HNSCC and uncovered differential gene expression signatures of therapeutics and prognostics potential [11, 12, 18–25]. Identification of altered gene expression signature in cancer will help in identifying key biological pathways, leading to better understanding of the underlying molecular mechanism of the disease and can be use in precise therapy selection in the HNSCC management [26–28].

Role of tobacco in the pathological process has been analysed in recent studies and found genes, which are frequently mutated and abruptly expressed, possibly due to tobacco exposure [29–31]. Despite these studies, our understanding of underlying molecular mechanisms influenced by tobacco in HNSCC patients is limited. Hence, there is need of uncovering key genes and its molecular mechanism in Tobacco induce and non-Tobacco HNSCC tumors. Therefore, in the present study differential gene expression analysis of tobacco smoking (we term as TOB) and non-smokers (we term as N-TOB) in TCGA HNSCC data set was performed for stratifying tobacco dependent and independent biological networks and functional pathways.

## RESULTS

### Detection of differentially expressed genes

**Figure 1:**
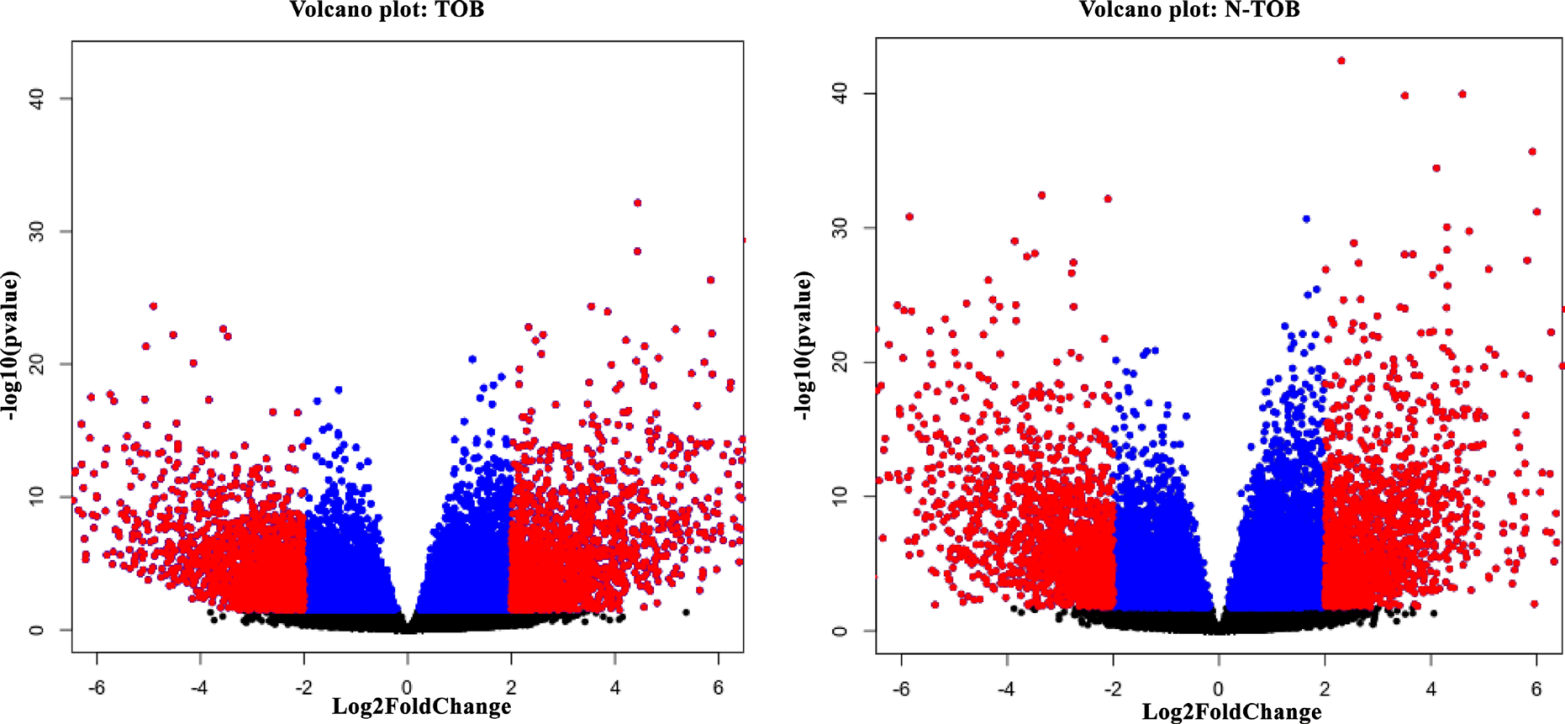
(**A**) Volcano plot showing gene expression significantly (FDR<0.05; LogFC>2) altered (highlighted in red colour) in TOB sample. (**B**) Volcano plot showing genes significantly (FDR<0.05; LogFC>2) altered in expression, highlighted in red colour in N-TOB patients.

RNA-seq analysis of 86 matched Tumor normal pairs of HNSCC have identified total 5968 upregulated & 5698 downregulated genes in habituated (tobacco smokers) tumors; and in non-Habituated tumors (non-tobacco users), total 6698 & 5896 genes showed up and downregulation respectively with *p-*value <0.1. Filtering based on fold change (*padj*(FDR)<0.05, Log2Foldchange>1) showed total total 6371 and 7003 DEGs in TOB and N-TOB samples respectively. Further filtering was done to consider genes which shows significant (*padj*(FDR)<0.05, Log2Foldchange>2) expression changes between Tumor and control samples. Total 2194 and 2073 genes were observed to show significantly altered expression in TOB and N-TOB tumors respectively (Figure 1, Supplementary Tables 1, 2). Among DEGs, 954 genes were up regulated and 1240 were down-regulated in TOB tumors. In case of N-TOB tumors, 890 and 1183 genes were found be up and downregulated respectively.

Additionally, Principal Component Analysis was performed using the PCA function from the sklearn Python module. Prior to performing PCA, the raw gene counts were normalized using the logCPM method, filtered by selecting the 2500 genes with most variable expression, and finally transformed using the Z-score method (Supplementary Figure 1).

**Figure 2:**
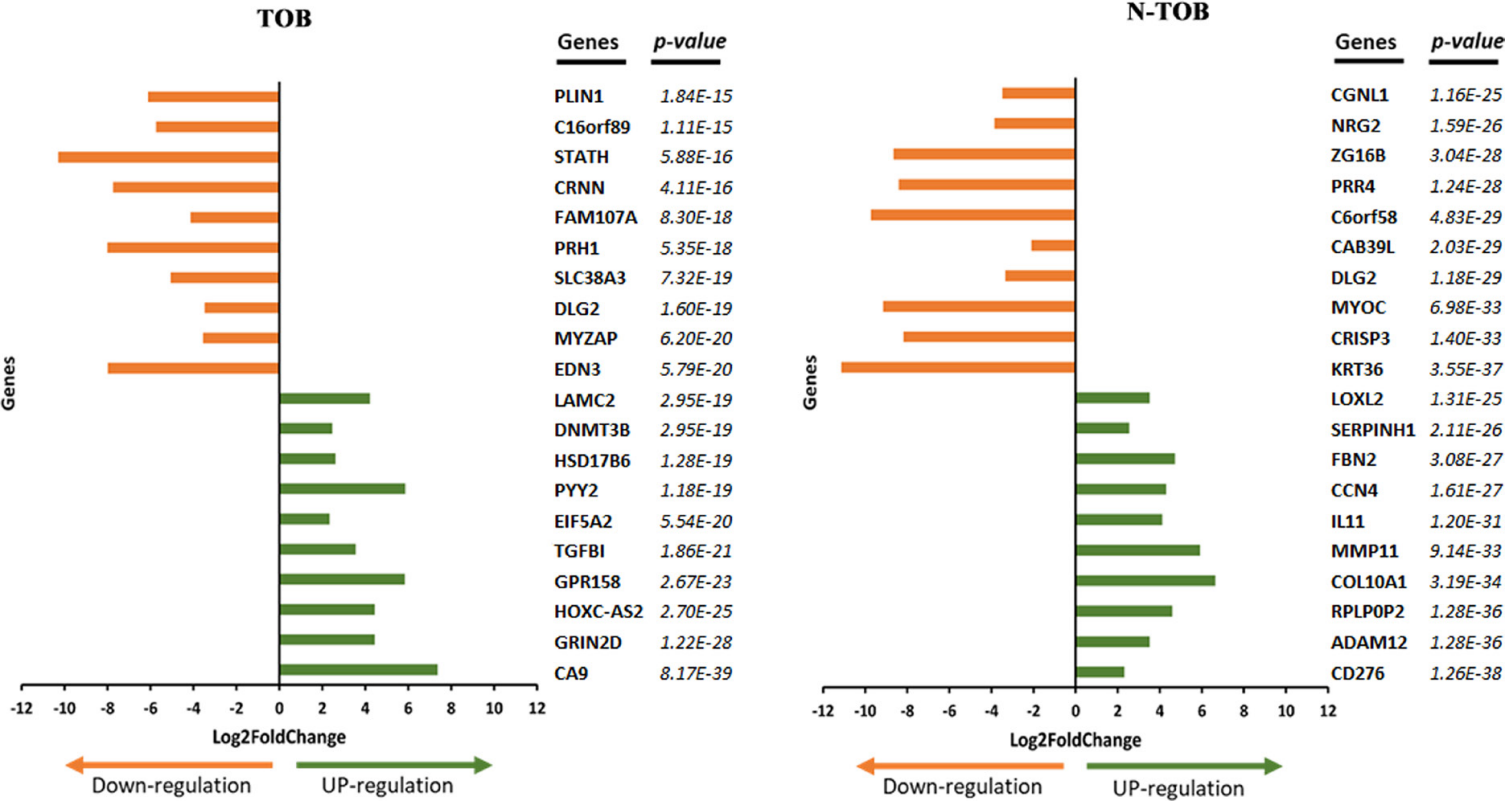
Top 20 DEGs identified in TOB and N-TOB tumors. Genes were ranked based on *p-*values ≤ 0.05 and adjusted false discovery rate using the Benjamini–Hochberg procedure.

A list of the top 20 most significantly up- and down-regulated DEGs of TOB and N-TOB tumors was shown in Figure 2. The most significant upregulated genes were CA9, GRIN2D, HOXC-AS2, GPR158, TGFBI etc. and genes i. e. EDN3, FAM107A, MYZAP, DLG2, SLC38A3, PRH1 etc. were observed to be most significant downregulated genes in TOB tumors. In N-TOB tumors, most significant upregulated genes were i. e. CD276, ADAM12, RPLP0P2 etc. and downregulated were KRT36, CRISP3, MYOC etc.

**Figure 3:**
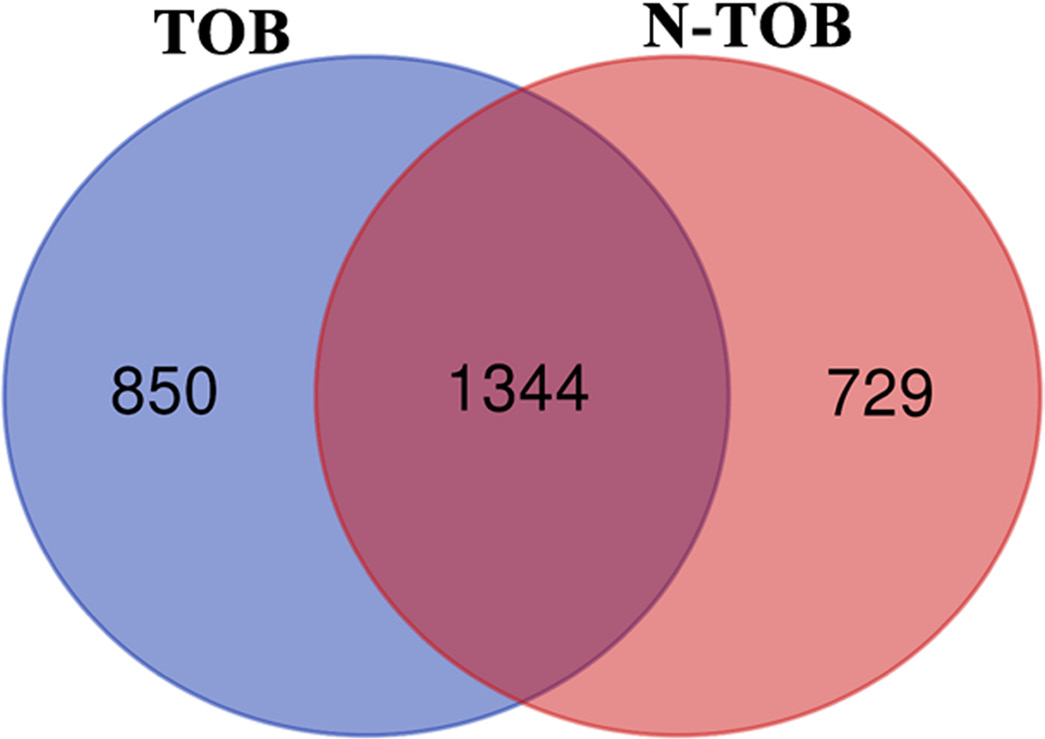
Venn diagram showing DEGs common and unique between Tobacco and non-Tobacco patients. Genes highlighted in blue and pink red are showed altered expression in Tobacco and non-Tobacco patients only; and genes highlighted in dark red are observed to be altered in both Tobacco and non-Tobacco patients.

In order to identify common Transcriptional signature shared between Tobacco smokers and non-tobacco smoker’s patients, comparison of DEGs between the two categories was performed. Of the total DEGs, 1344 genes were observed to be altered in both categories whereas, 850 and 729 genes were observed to be unique in TOB and N-TOB tumors respectively (Figure 3).

### Gene ontology and pathway enrichment analysis

**Figure 4:**
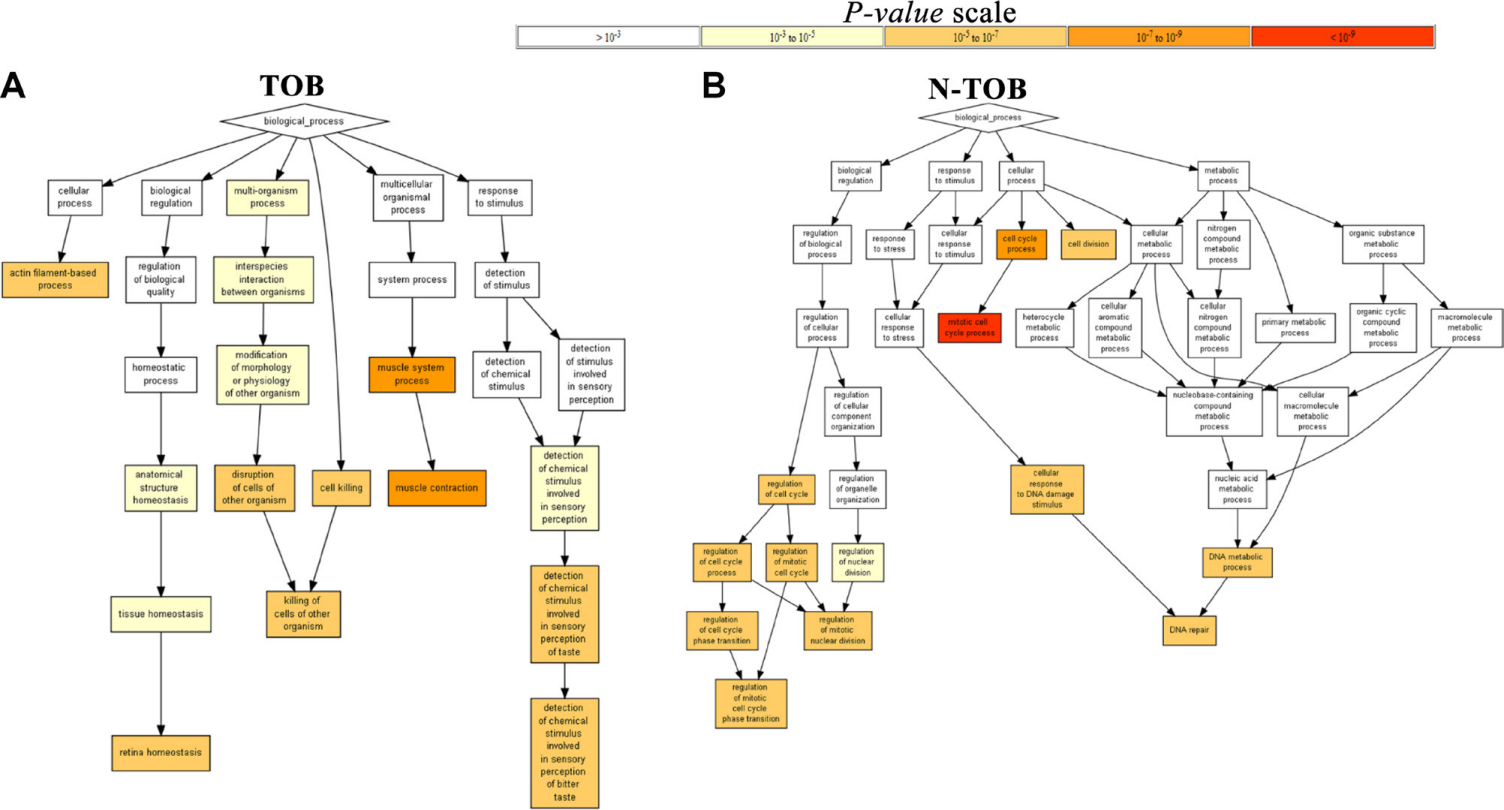
Gene Ontology Enrichment Analysis Results. The figure contains interactive charts displaying the results of the Gene Ontology (GO) enrichment analysis generated using GOrilla. The x-axis indicates the enrichment score for each term. Significant terms are highlighted in bold.

In order to gain insights into the biological roles of the DEGs in HNSCC, we performed Gene ontology enrichment (*p* value<0.05) analysis using Gorilla software. The GO terms for biological process was found to be enriched in muscle contraction (GO:0006936; *P = 2.14E-7, P = 1.95E-9*) and retinal homeostasis (GO:0001895; *P = 3.81E-6, P = 4.36E-7*) in both TOB and N-TOB tumors (Figure 4).

**Figure 5:**
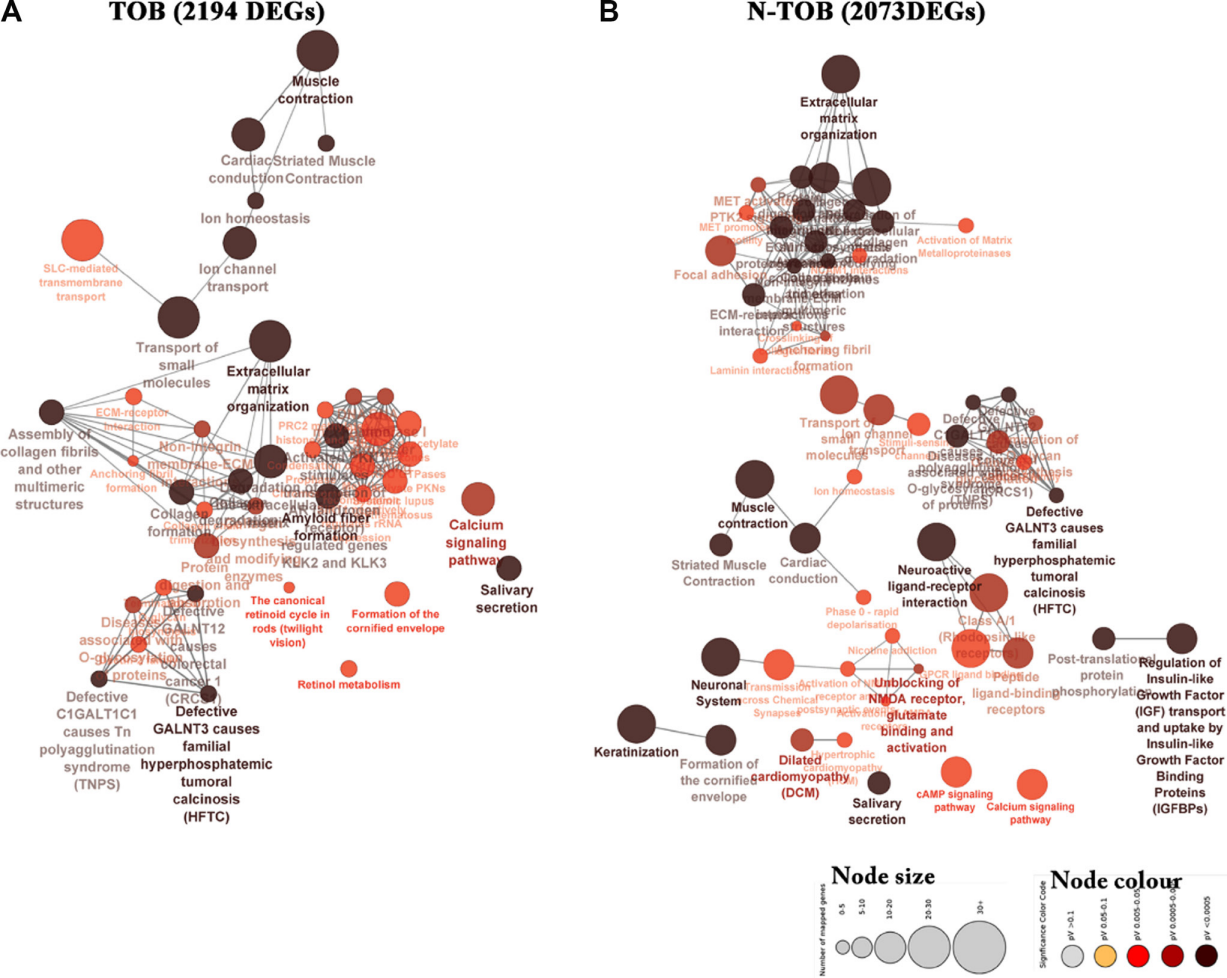
Network representing enriched pathways integrated KEGG and Reactome pathways of both TOB and N-TOB tumors. Highest significance of enriched pathway was obtained using advanced statistical settings such as Hyper-geometric (right-handed) enrichment distribution tests, *p-*value < 0.05, and Bonferroni adjustment. The size and colour represents number of DEGs involved and enrichment significance respectively- deeper the colour, the higher the enrichment significance.

Further, biological significance for the DEGs was evaluated using KEGG and Reactome pathway enrichment analysis (*p-*value ≤ 0.05). The most significant pathway based on right sided hypergeometric test and bonferroni (pV) adjustments was muscle contraction (*pV = 2.55E-13, pV = 9.09E-16*), Extracellular matrix organization (*pV = 9.28E-09, pV = 1.70E-18*) and salivary secretion (*pV = 1.83E-07, pV = 5.76E-5*) in both TOB and N-TOB tumors (Figure 5). In addition to this, pathway related to Neuroactive ligand-receptor interaction (*pV = 1.49E-09, pV = 1.70E-18*) was observed to be enriched more in N-TOB tumors (Figure 5).

### Protein-protein (PPI) interaction analysis of DEGs of Tobacco and non-Tobacco patients

**Figure 6:**
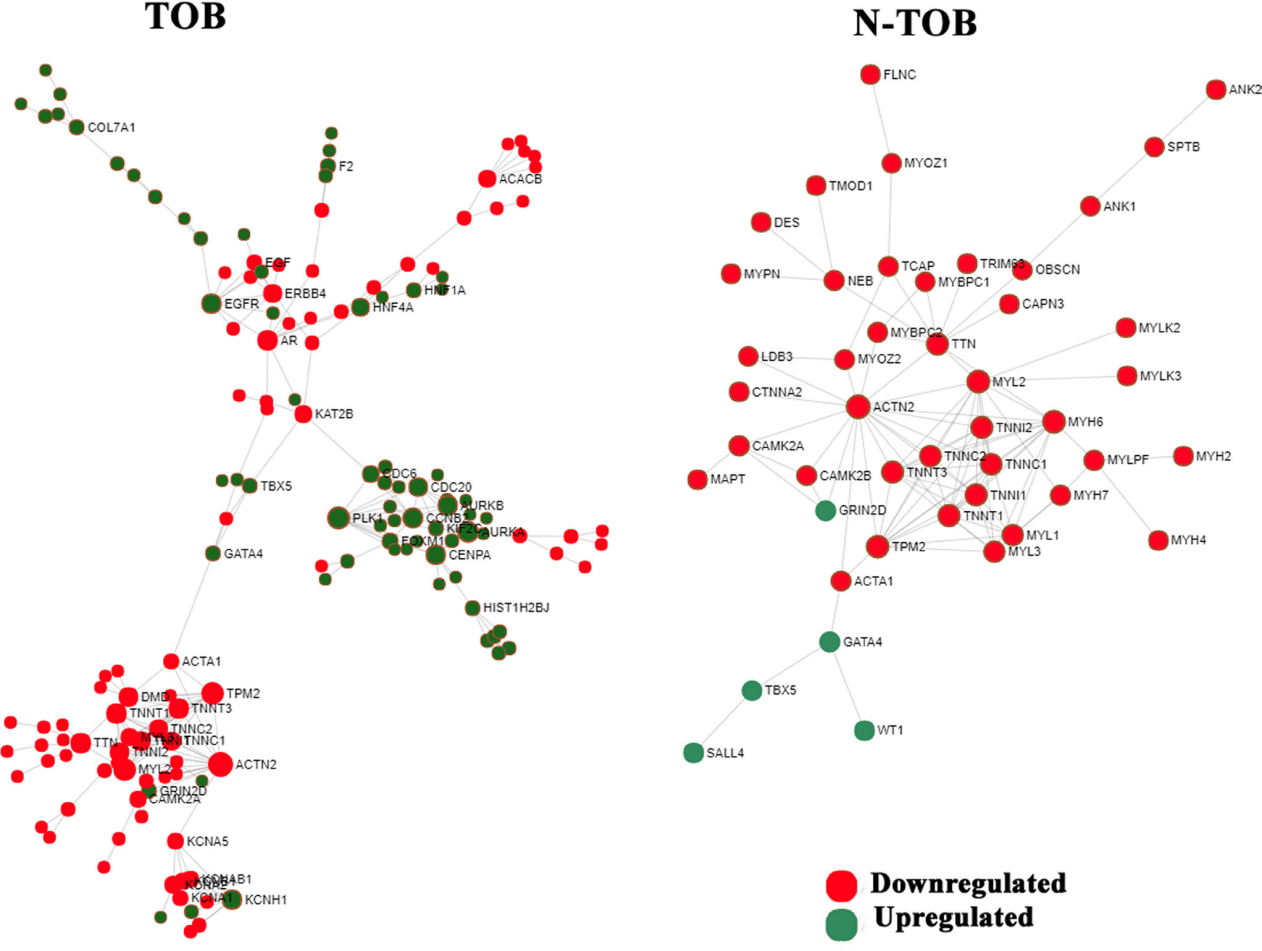
Network based meta-analysis of hub genes. Zero-order interaction network of DEGs obtained from RNA-seq data using force-directed algorithm with Fruchterman-Rengold layout; green nodes represents over-expressed and red nodes represents under-expressed genes.

The PPI network of DEGs were constructed for both TOB and N-TOB tumors by mapping genes onto STRING interactome database with higher confidence score cutoff of 0.900. The PPI network consists of 1552 nodes and 2576 edges in Tobacco DEGs, whereas N-TOB DEGs were observed to have 1913 nodes and 2969 edges. Hub genes were identified based on the number of interacting partners they have in the biological network. It was found that, genes such as ACTN2, PLK1, TPM2, MYL2, AURKA, AR, CCNB2, EGFR, TTN, CENPA, were observed to be top 10 hub genes in TOB tumors, whereas N-TOB tumors showed ACTN2, MYH6, MYL2, TPM2, TTN, TNNI2, TNNT3, TNNI1, TNNT1, TNNC1 as hub genes in the network (Figure 6). Of these, hub genes ACTN2, MYL2, and TPM2 were observed to be downregulated in both the cases. However, upregulated hub genes i. e. PLK1 *(Degree = 13; Betweenness = 1898)*, AURKA *(Degree = 11; Betweenness = 1216)*, AR *(Degree = 10; Betweenness = 4872)*, CCNB2 *(Degree = 10; Betweenness = 1045)* and EGFR *(Degree = 9; Betweenness = 2234)* were observed in Tobacco tumors.

### miRNA enrichment analysis

miRNA Enrichment results were generated by analyzing the up-regulated and down-regulated gene sets using Enrichr. Significant results was obtained using cut-off of *p-*value<0.05 after applying Benjamini-Hochberg correction (FDR<0.05). The most enriched miRNAs terms were hsa-miR-29 family, which are regulating DEGs in both TOB and N-TOB tumors (Table 1). Interestingly, TOB has more enriched miRNAs terms than N-TOB tumors. microRNA-29a/b/c were enriched miRNAs terms and observed to be upregulating 25, 33 and 24 target genes, in the TOB tumors respectively. In case of N-TOB, only hsa-miR-29b-3p* were found as enriched miRNA, possibly involved in upregulation of 20 target genes. Addition to this, TOB tumors has other enriched miRNAs *i. e.* hsa-miR-215-5p*, hsa-miR-193b-3p*, hsa-miR-192-5p*, hsa-miR-124-3p*, hsa-miR-615-3p*, hsa-miR-26b-5p*, hsa-miR-92a-3p* which regulates 75, 77, 81, 70, 50, 82 and 65 target genes respectively.

**Table 1:** miRNA enrichment analysis results

Rank	miRNA	*P-*value	FDR	Target	Rank	miRNA	*P-*value	FDR	Target
TOB					N-TOB				
1	hsa-miR-215-5p*	6.08E-25	1.62E-21	75 upregulated	1	mmu-miR-29a-3p*	0.000002	0.004102	9 upregulated
2	hsa-miR-193b-3p*	5.10E-23	6.78E-20	77 upregulated	2	mmu-miR-29b-3p*	0.000004	0.00419	9 upregulated
3	hsa-miR-192-5p*	2.30E-21	2.03E-18	81 upregulated	3	hsa-miR-29b-3p*	0.00001	0.006585	20 upregulated
4	hsa-miR-29b-3p*	1.94E-14	1.29E-11	33 upregulated	4	mmu-miR-124-5p	0.002887	1	3 downregulated
5	hsa-miR-29a-3p*	1.34E-08	7.12E-06	25 upregulated	5	mmu-miR-1897-5p	0.017333	1	4 downregulated
6	hsa-miR-29c-3p*	2.73E-08	1.21E-05	24 upregulated	6	mmu-miR-3098-5p	0.019988	1	2 downregulated
7	hsa-miR-124-3p*	6.95E-08	2.64E-05	70 upregulated	7	mmu-miR-467e-5p	0.019988	1	2 downregulated
8	hsa-miR-615-3p*	8.43E-08	2.80E-05	50 upregulated	8	mmu-miR-467h	0.019988	1	2 downregulated
9	hsa-miR-26b-5p*	3.43E-07	1.01E-04	82 upregulated	9	mmu-miR-543-3p	0.023748	1	3 downregulated
10	hsa-miR-92a-3p*	1.06E-06	2.83E-04	65 upregulated	10	hsa-miR-502-5p	0.037752	1	5 downregulated

The table displaying the results of the miRNA enrichment analysis generated using Enrichr. Every row represents a miRNA; significant (*p-*value < 0.05; FDR < 0.005) miRNAs are highlighted in bold. Displays results generated using the miRTarBase library.

### Potential biomarker

**Figure 7:**
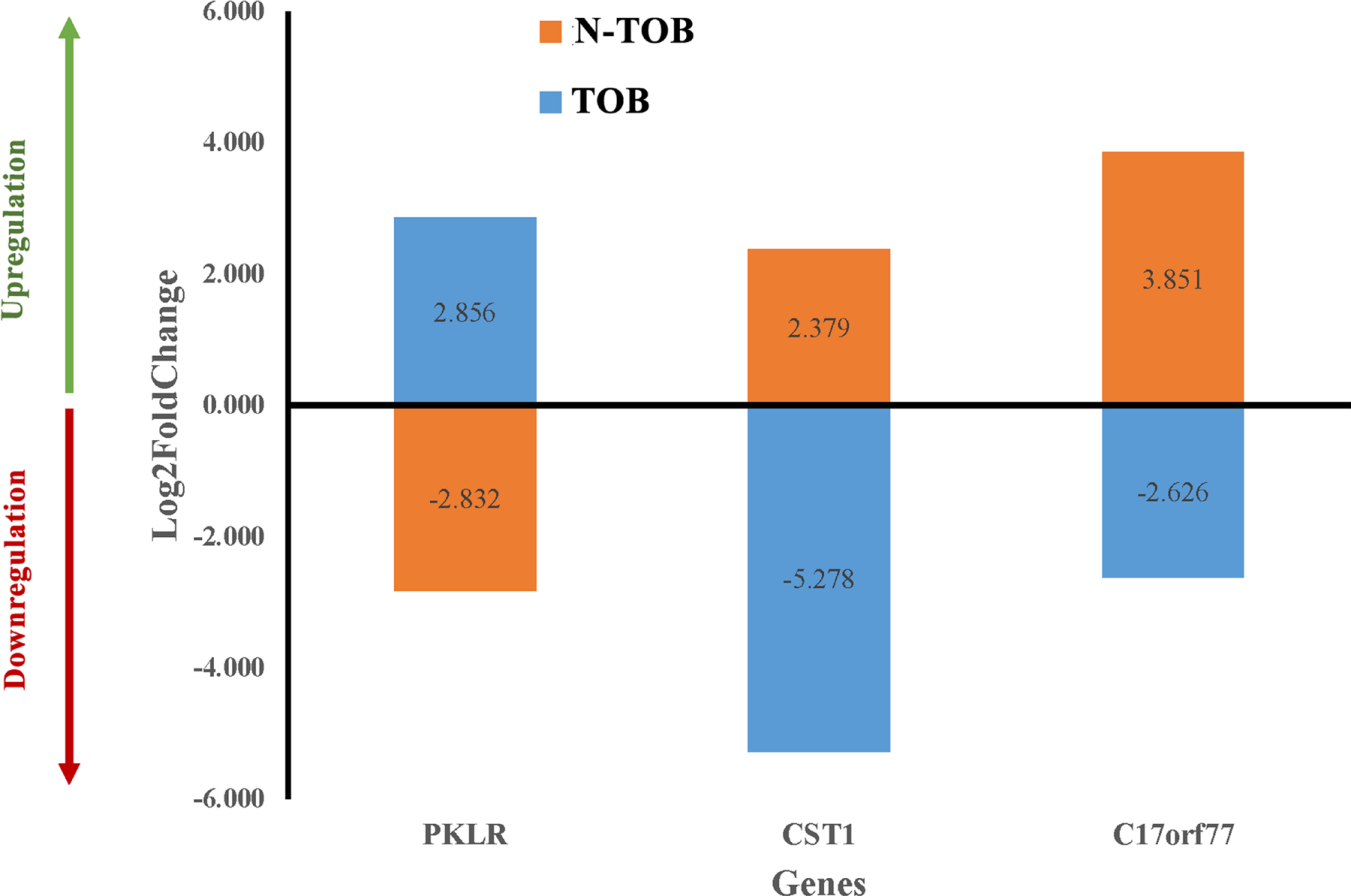
Differential gene expression pattern of key genes in both TOB and N-TOB tumors. Log2fold change values of genes showing opposite regulation of the same gene in between TOB and N-TOB tumors.

In order to find candidate genes, which behave differently and can be used to separate TOB tumors from N-TOB tumors, Log2fold change of gene of both tumors were compared. Surprisingly, three genes namely PKLR1, CST1 and C170rf77 were observed to expressed differently in both TOB and N-TOB tumors, which means the same genes is upregulating in one category, whereas downregulating in another categories of HNSCC tumors (Figure 7).

### External validation of the DEGs in non-matched tumors

Further, the consistency of the identified altered gene expression profile was checked in other non-matched tumors samples of tobacco smoking and non-smoking patients. For this, non-matched tumors samples having tobacco smoking history and those who never smoked, were searched in TCGA database. Total 224 and 151 samples of smoking habit and non-smokers patients were selected respectively. For DGE analysis, the normal control from the matched pairs (as used in the above analysis) were taken and process further using same pipeline (see method).

Results showed, nearly 83% of matched tumor DEGs (*padj*(FDR)<0.05, Log2Foldchange>1), were also observed to be deregulated in non-matched tumors, whereas 88% (1722 of total 2194 in TOB; 1643 of total 2073 in N-TOB) concordance was observed with fold change 2 (Log2Foldchange>2) (Supplementary Tables 3, 4). In addition to similar DEGs pattern, the top differentially expressed genes and three candidate genes of matched pairs were also found to be abruptly expressed in non-matched tumors. The expression pattern of candidate genes i. e. PKLR1, CST1 and C170rf77 in non-matched samples showed similar pattern with little low fold change as compared to matched tumors (Supplementary Figure 2).

## DISCUSSION

Tobacco usage is a major factor in the development of various cancers including Head and neck squamous cell carcinoma (HNSCC). Present study attempted to identify differential expressed genes (DEGs) signature and its underlying possible molecular mechanism in 86 matched tumor normal pairs of 43 HNSCC patients having Tobacco smoking (17 samples) and non-smoking (26 samples) history. Findings was further validated on 375 non-matched HNSCC tumors having tobacco smoking history (tobacco smoking = 224; non-smokers = 151) available in the TCGA database. Total, 2194 (954 up regulated and 1240 down regulated) and 2073 (890 up regulated and 1183 down regulated) genes were observed to be differentially expressed in TOB and N-TOB tumors respectively (Figure 1).

In order to uncover the biological roles of the DEGs in each TOB and N-TOB tumors, we performed a gene ontology (GO) enrichment analysis. GO terms related to muscle contraction, retinal homeostasis were enriched in TOB tumors; whereas cell cycle, cell division and DNA repair in N-TOB tumors were observed to be highly enriched biological process. Genes related to muscle contraction has been reported to be altered in Oral squamous cell carcinoma (OSCC) and suggests the presence of myofibroblasts in tumor stoma of patients with lymph node involvement [32].

Pathway enrichment analysis identified pathways related to extracellular matrix organization, muscle contraction, calcium signaling and salivary secretion to be the most significantly enriched pathways in both categories. In case of N-TOB patients, pathways of Neuroactive ligand-receptor interaction were found to be enriched. Extracellular matrix organization genes regulates cell proliferation, adhesion, differentiation, death and alteration leads to tissue fibrosis and cancer [33], whereas muscle contraction pathways were reported to be deregulated in lymph node metastasis OSCC [32]. Deregulation of the calcium signal is reported to be involved in tumor initiation, angiogenesis, progression and metastasis [34, 35] and a promising pathway for therapeutics strategy designing [26].

In recent years, effects of etiological factors such as lifestyle, diet, environment and exposure on molecular pathogenesis has been well studied due to emergence of the field i. e. molecular pathological epidemiology (MPE) [36, 37]. MPE is an integrative field, which incorporates molecular pathology into epidemiologic research and dissect the link between heterogeneous etiological factors such as environmental, dietary habits, microbiota, lifestyle, exposure and genetic factors with tumor initiation and progression in cancers of breast, prostate, lung and colorectal [38–42]. Moreover, the present study identified calcium-signaling pathway to be enriched in TOB tumors, and may have association of smoking exposure with molecular pathogenesis of HNSCC. Calcium signaling is known to be affected by etiologic factors such as cigarette smoke exposure and results into increase calcium levels in cells and plays an important role in proliferation, migration, invasion or tumor growth and metastasis in cancers including drug resistance of pancreatic cancer [43–47]. Hence, in HNSCC it is possible that cigarette smoke increases intracellular calcium levels and affects normal cellular functions by promoting proliferation, motility, invasion and survival. Therefore, targeting calcium-signaling pathway in future HNSCC research, will provide detailed molecular insights of smoking related cancers and help in developing more effective treatment strategies in HNSCC. Neuroactive ligand-receptor interaction pathway has been reported to be implicated in many cancer types [26, 48–50] including OSCC [51] and represented promising candidates for therapeutic intervention in OSCC patients [26]. Therefore, targeting Neuroactive ligand-receptor interaction pathway in N-TOB tumors may open new avenues in HNSCC therapeutics intervention.

PPI network analysis elucidated detailed interaction among TOB and N-TOB DEGs and highlighted top 10 centrality hub genes. Interestingly, MYH6 (*Degree = 13; Betweenness = 183*) hub gene is observed to be downregulated only in N-TOB tumors, hence suggested to play important role in tobacco independent disease development and progression. Role of myosin heavy chain 6 (MYH6) gene has earlier been associated in familial dilated cardiomyopathy [51, 52]. However, some study using RNAi *in vivo* screen identified MYH6 frequently altered in HNSCC MOC lines [53] and consider as novel putative cancer genes [54]. EGFR gene was observed to be upregulated in TOB tumors only, hence may be possibly involved in tobacco mediated carcinogenesis. Previously it was reported [55] that EGFR was overexpressed in about 30% of human epithelial tumors including HNSCCs [55, 56]. Ansell et al. 2016 found that the amount of EGF had a determinant function in cell proliferation and the response to treatment of cetuximab in tongue cancer, and emerged as a potential predictive biomarker of poor cetuximab response [57]. High level of EGFR enhanced proliferation, promote tumor growth leading to poor prognosis [58–60] and resistant to radiotherapy [61–63].

The prominent nodes with lowest *p-*values signify presence of muscle contraction pathways and associated altered expression of genes such as, TTN, TNNT3, TNN2 and MYL2 in both TOB and N-TOB tumors. This is in concordance with earlier microarray based gene expression profiling study, which revealed a distinction signature pattern belonging to muscle contraction pathway [32]. Furthermore, different studies have already proved the presence of myofibroblasts of tumor stroma by gene expression analysis in lymph nodes establishing their role in metastatic migration, invasion and association with poor survival rate in OSCC patients [64–66]. Troponin (TNN) gene family reported to be altered in TSCC and might serve as future clinical prognostic marker for TSCC [67]. These genes might play a regulatory role in control of cellular locomotion, cytoplasmic streaming, and cytokinesis in non-muscle cells. Further studies are warranted to elucidate the role of muscle related genes in OSCC carcinogenesis.

Polo-like kinase 1 (PLK1) encodes the protein which is important regulators of the cell cycle and cell division. PLK1 has been reported to be upregulated in various cancers [67, 68] and their expression level are associated with a poor prognosis and a lower overall-survival in many cancers [69–71] including HNSCC [72]. Recent study has demonstrated this as a promising target for chemopreventive treatment of preneoplastic cells, and could be applied to prevent HNSCC and local relapses [72, 73]. Aurora kinase A (AURKA) has been implicated in numerous types of cancer [72–78] and reported to promotes cell migration & invasion [79] tumor progression in patients with laryngeal squamous cell carcinoma (LSCC) [80]. Both PLK1 and AURKA hub genes were observed to be upregulated, which is in concordance with the above earlier reports.

Interestingly, three genes i. e. PKLR, CST1 and C17orf77 were observed to expressed differently in the TOB and N-TOB tumors, means there is upregulation in TOB, whereas downregulation in N-TOB and vice versa. Surprisingly, role of PKLR gene in HNSCC cancer not well studied. However, recent study, have demonstrated that PKLR promotes colon cancer cell metastatic colonization of the liver by increasing glutathione synthesis, which is the primary endogenous antioxidant [80, 81]. CST1 gene encodes Cysteine proteases (CST1) enzymes, generally involve in protein degradation, which is associated with a diversity of diseases and facilitates the development and progression of cancer cells [82–84]. Cystatins (CST1) play important roles in tumor invasion and metastasis in colorectal cancer [84–86]. Recent study reported that elevated expression of CST1 may promotes breast cancer progression and predicts a poor prognosis [87]. Role of Chromosome 17 Open Reading Frame 77 (C17ORF77) gene in cancer has not been well studied yet. Therefore, these three genes will be gene of interest in future studies for better understanding of molecular insight of tobacco habituated and non-habituated associated HNSCC.

Additionally, the present study also identified enriched riboswitches i. e. miRNAs regulating their target genes for understanding regulatory cascade of tobacco and non-tobacco induced carcinogenesis. microRNA-29a/b/c were observed to be the most enriched miRNAs (*P-*value < 0.05) across both TOB and N-TOB categories, as it regulate genes, which are detected to be upregulated in the present study. In earlier studies, miR-29 family members (i. e., miR-29a, miR-29b, and miR-29c) are reported to be frequently downregulated in many cancers [87–91] including their antitumor functions in HNSCC [91–93]. Therefore, downregulation of *miR-29* family leading to loss of tumor suppressor activity, which subsequently results in the upregulation of several oncogenic genes in HNSCC. TOB tumors possesses other enriched miRNAs i. e. hsa-miR-215-5p*, hsa-miR-92a-3p*, hsa-miR-26b-5p* etc. Among these, the largest number of upregulated i. e. 82 genes were found associated with miR-26b, which is reported to be downregulated in various cancers, including OSCC, indicating its tumor suppressive nature in oral cancer progression [93, 94]. hsa-miR-26b-5p was also reported as tumor suppressors in cancers of oral cavity [2, 89] and suggested that, loss of tumor-suppressive miR-26a/b enhanced cancer cell migration and invasion in OSCC [2]. Therefore, identification of gene networks regulated by these tumor suppressor miRNAs may provide novel insights into designing therapeutic strategies in HNSCC.

## METHODS

### Identification of relevant datasets and differential expression analysis

**Figure 8:**
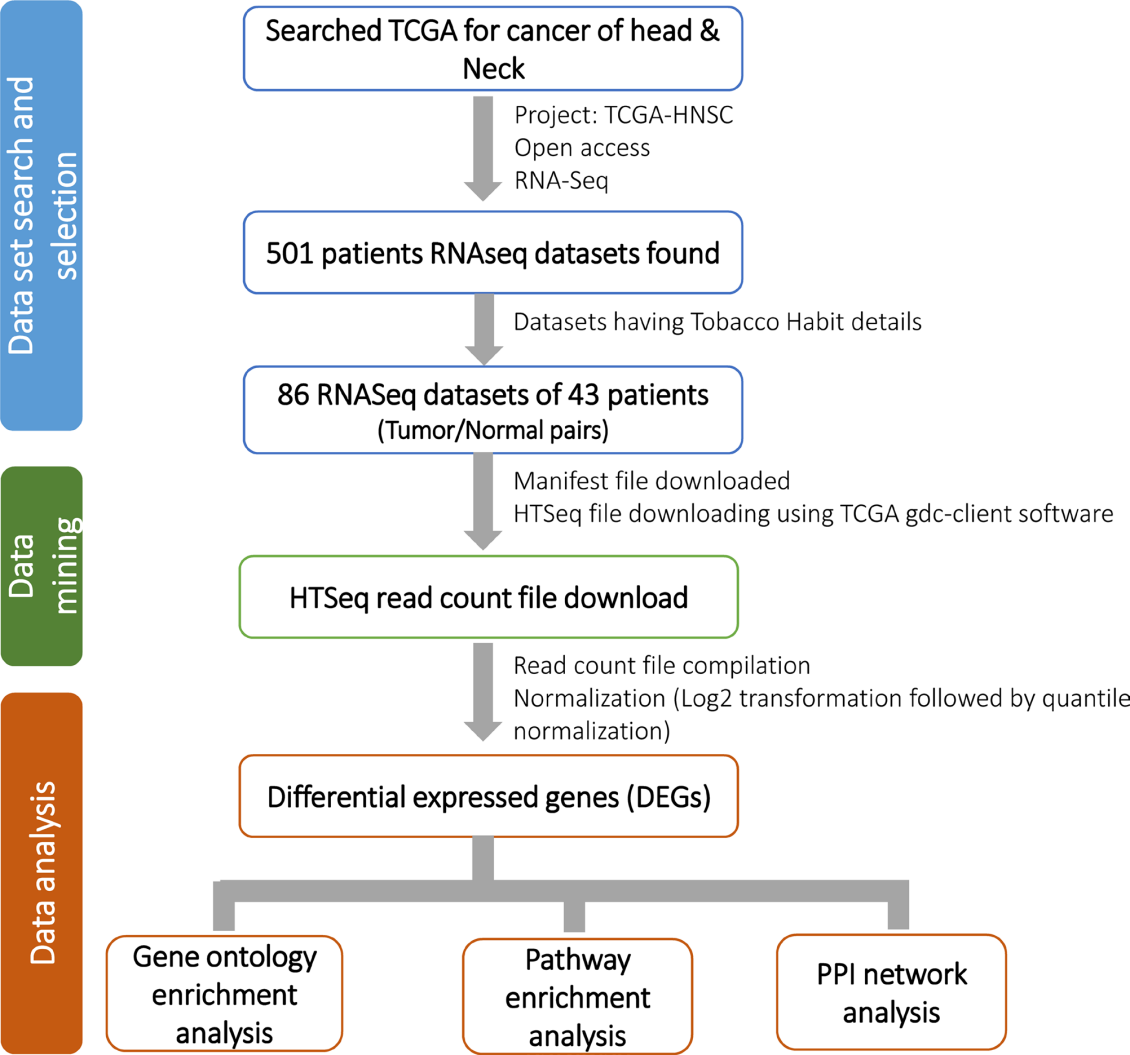
Detail workflow of the study. Study workflow consists of three components i. e. 1) data set search and selection of relevant data; 2) mining of the selected data (RNA-seq read count) from TCGA; and 3) data analysis which includes differential gene expression (DEGs) analysis and annotations.

RNA-seq data was retrieved from The Cancer Genome Atlas, TCGA-HNSC project for gene expression profiling of HNSCC patients for our study. Total 86 Tumor/Normal pairs samples of 43 HNSCC patients were found having tobacco habit details (Table 2). Of these, 34 sample pairs from 17 Tobacco habituated patients and 52 sample pairs from 26 non-habituated patients were considered for further analysis. Raw read counts (HTSeq read counts) of total 86 Tumor/Normal pairs were downloaded using TCGA GDC Data Transfer Tool (Figure 8). Raw read counts were normalized to log10-Counts Per Million (logCPM), followed by the application of a log10-transform using DESeq R package and differentially expressed genes (DEGs) were identified for each TOB and N-TOB tumor.

**Table 2:** Patients details including age, stage, Tobacco habit, alcohol consumption status, demography and gender shown in the table

Study Characteristic	No.
Age at diagnosis, years	
Mean ± SD	64.9 ± 14.5
Median	42
**Clinical stage**	
Stage I	2
Stage II	15
Stage III	8
Stage IVa	17
Not reported	1
**Tobacco habit (Smoking)**	
Yes	17
No	26
**Alcohol consumption status**	
Yes	22
No	19
Not reported	2
**Gender**	
Male	29
Female	14
**Demographic details**	
White	39
Asian	1
Black or african american	2
not reported	1

Matched Tumor/Normal pairs of each HNSCC patient was retrieved from TCGA database.

### Gene ontology (GO) and pathway enrichment analysis

In order to understand the biological significance of the DEGs, we performed Gene Ontology (GO) enrichment analysis using GOrilla tool [95] with *p-*value threshold of 0.05. Enriched pathways of DEGs were identified using KEGG and Reactome pathway databases integrated in ClueGO [95, 96] v2.3.5 plugin of Cytoscape 3.4.0 with *p-*value cut-off 0.05. Additional filtering was performed using advance statistical option such as Two-sided hypergeometric test for calculating importance of each term and Bonferroni step-down for *p-*value correction.

### PPI network construction

Protein-protein interaction (PPI) of analysis was performed for DEGs of both categories to identify key proteins and their significance in the complex biological systems. We used STRING interectome database [97] with high confidence score cut-off (0.900) and constructed PPI network of DEGs using NetworkAnalyst [98]. Further, filtering of zero-order network was performed to retain only seed proteins that directly interact with each other and finally highly interconnected hub nodes was identified based on two centrality measure such as degree and betweenness centrality.

### miRNA enrichment analysis of DEGs

microRNAs enrichment analysis was performed to identify regulatory cascade of DEGs of HNSCC. Two databases *viz.* TargetScan [98, 99] and MiRTarBase [100] integrated in Enrichr [100, 101] tool was used to retrieved enriched (*p-*value<0.05) riboswitches involve in transcriptional regulation of DEGs.

## CONCLUSIONS

In conclusion, we performed analysis of HNSCC RNA-seq data and identified key deregulated genes associated with functional pathways and biological networks, which may be contributing in Tobacco dependent and independent carcinogenesis of the disease. Pathway analysis identified key DEGs involves in calcium signaling, and suggests playing key role in tobacco dependent molecular pathogenesis in HNSCC. Our findings suggest that, three genes i. e. PKLR, CST1 and C17orf77 may hold putative role in both pathogenesis of smoking and non-smoking related HNSCC tumors and can be consider as potential biomarker for separating these tumors from each other. The identified differentially expressed genes can be integrated in multiple biological pathways and provide improved understanding of key molecular mechanisms in smoking and non-smoking associated HNSCC tumors, and be useful in precision therapy selection. However, further research is needed to understand influence of smoking and other etiological factors such as environment, lifestyle, diet, alcohol consumption and HPV infection on the molecular pathogenesis of the disease.

## SUPPLEMENTARY MATERIALS










