# Supplementary material for: Differential gene expression analysis of HNSCC tumors deciphered tobacco dependent and independent molecular signatures
Source: Oncotarget. 2019 Oct 22;10(58):6168–83. doi: 10.18632/oncotarget.27249 (PMC6817442; doi:10.18632/oncotarget.27249)
Supplement: Supplementary file 1 [file oncotarget-10-6168-s001.pdf]

Differential gene expression analysis of HNSCC tumors deciphered tobacco dependent and independent molecular signatures

SUPPLEMENTARY MATERIALS

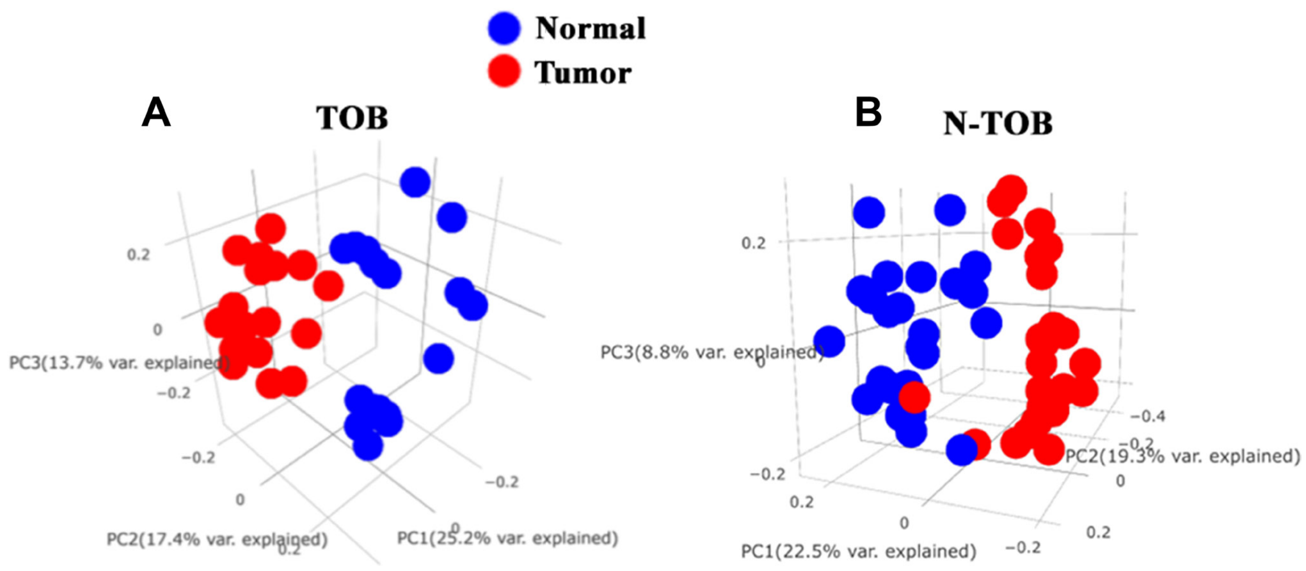

Supplementary Figure 1: Principal Component Analysis results. The figure displays an interactive, three-dimensional scatter plot of the first three Principal Components (PCs) of the data. Each point represents an RNA-seq sample. Samples with similar gene expression profiles are closer in the three-dimensional space. Point highlighted with red and blue colour are Tumor and normal control respectively.

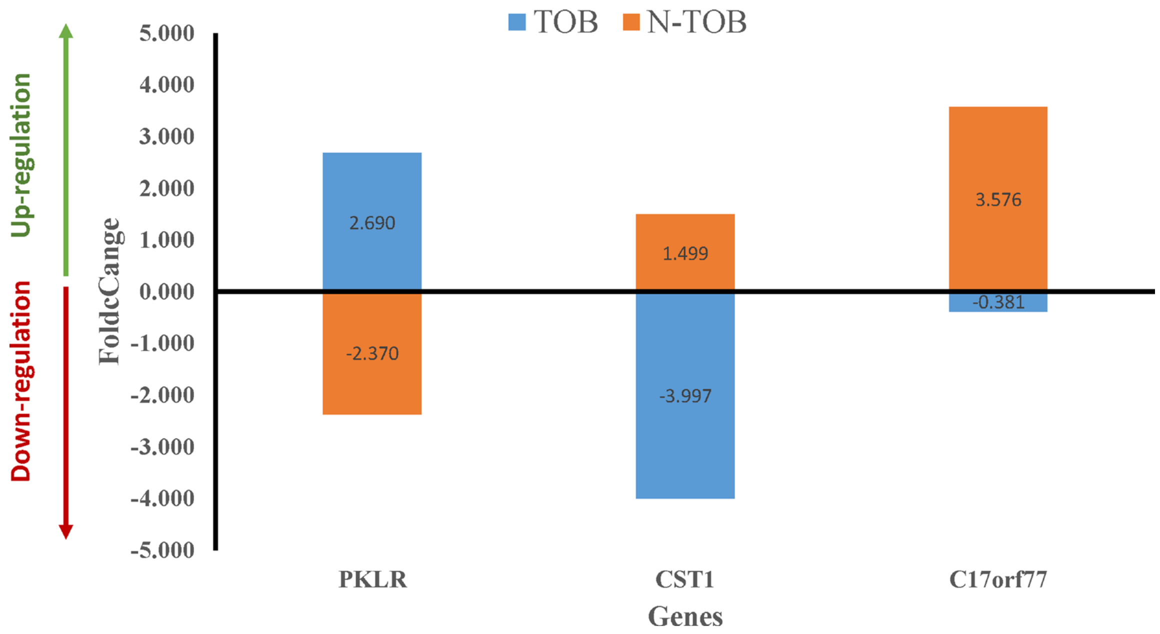

Supplementary Figure 2: Validation of differential gene expression pattern of key genes in both TOB and N-TOB tumors on non-matched tumors. Foldchange change values of genes showing opposite regulation of the same gene in between TOB and N-TOB tumors.

Supplementary Table 1: List of DEGs (padj (FDR)<0.05, Log2Foldchange>2) identified in matched tumors of smokers patients. See Supplementary Table 1

Supplementary Table 2: List of DEGs (padj (FDR)<0.05, Log2Foldchange>2) identified in matched tumors of non-smokers patients. See Supplementary Table 2

Supplementary Table 3: List of DEGs (padj (FDR)<0.05, Log2Foldchange>1) identified in non-matched tumors of smokers patients. Genes highlighted with yellow background is the also identified in matched tumors (padj (FDR)<0.05, Log2Foldchange>1). See Supplementary Table 3

Supplementary Table 4: List of DEGs (padj (FDR)<0.05, Log2Foldchange>1) identified in non-matched tumors of non-smokers patients. Genes highlighted with yellow background is the also identified in matched tumors (padj (FDR)<0.05, Log2Foldchange>1) See Supplementary Table 4
